# Standardized Ileal Digestibility of Amino Acids in Hybrid Rye Ground to Two Particle Sizes and Fed With or Without Multienzyme Supplement to Young Growing Pigs

**DOI:** 10.1111/jpn.14053

**Published:** 2024-10-23

**Authors:** Jichen Song, Claus H. Heuer, Rob Patterson, Charles M. Nyachoti

**Affiliations:** ^1^ Department of Animal Science University of Manitoba Winnipeg Manitoba Canada; ^2^ KWS, Lochow GMBH Bergen Germany; ^3^ CBS BioPlatforms Inc. Calgary Manitoba Canada

**Keywords:** digestibility, enzyme supplementation, hybrid rye, particle size, pigs

## Abstract

A newly developed hybrid rye with enhanced ergot resistance has potential as an alternative energy source for pigs. An experiment was conducted to evaluate the effects of particle size (PS) and multienzyme supplement (MES) on the apparent total tract digestibility (ATTD) of energy and nutrients and the standardized ileal digestibility (SID) of amino acids (AA) in hybrid rye fed to growing pigs. Bono and Gatano, two hybrid rye varieties, were ground to either a coarse (1111 and 1210 μm) or a fine (594 and 717 μm) PS using a hammer mill. Eighteen ileal‐cannulated barrows (initial BW = 18.2 ± 1.0 kg) were randomly assigned to 1 of 9 dietary treatments in a replicated 9 × 3 incomplete Latin square design to give six observations per treatment. Among the nine experimental diets, eight featured two hybrid rye varieties (Bono or Gatano), either coarsely or finely ground, as the only source of AA with or without MES, while an N‐free diet was used to estimate the endogenous losses of AA at the distal ileum. All diets contained titanium dioxide as an indigestible marker. Each period lasted 9 days, with the first 5 days being for adaptation followed by 2 days each for faecal and ileal digesta collection. Data were analysed using the MIXED procedure of SAS, with the final model having variety, PS, MES, their two‐way interactions, and a three‐way interaction. No interactions were noted except that the apparent ileal digestibility and SID of crude protein (CP) and most AA in Gatano had greater effects of MES than Bono (Variety × MES; *p* < 0.05). The ATTD of dry matter (DM), gross energy (GE) and CP were greater (*p* < 0.05) in Bono diets than in Gatano diets. Finely ground hybrid rye diets had higher (*p* < 0.05) ATTD of neutral detergent fibre assayed with a heat stable amylase and expressed inclusive of residual ash (NDF) and ether extract compared to coarsely ground hybrid rye diets. Dietary MES supplementation increased (*p* < 0.05) the ATTD of DM, GE, CP, NDF, acid detergent fibre expressed inclusive of residual ash, and ash in hybrid rye diets. In conclusion, the nutrients digestibility of hybrid rye can be affected by its variety. PS reduction improved the nutrient digestibility in hybrid rye, and MES supplementation improved the energy and AA digestibility in hybrid rye fed to growing pigs.

## 1 Introduction

A recently developed hybrid rye demonstrates reduced susceptibility to ergot contamination while maintaining high yield levels (Miedaner et al. 2021). Additionally, this new hybrid rye has metabolizable energy values comparable to those of barley and sorghum, thereby positioning it as a potential alternative energy source for swine nutrition (McGhee and Stein 2020). Nevertheless, rye is characteristically high in non‐starch polysaccharides (NSP), primarily arabinoxylans, cellulose and β‐glucans, which can increase the digesta viscosity and consequently affect the nutrient digestibility in pigs (Knudsen 1997; Salmenkallio‐Marttila and Hovinen 2005).

The utilization of multiple enzyme complex has been empirically substantiated to outperform the use of single enzyme in the depolymerization of NSP in cereal grains, thereby enhancing nutrient digestibility and growth performance in pigs when fed a variety of feedstuffs (Omogbenigun, Nyachoti, and Slominski 2004; Emiola et al. 2009). Moreover, a decrease in particle size (PS) can improve the nutritional value of feed ingredients, as it enlarges the surface area available for enzymatic interaction during the digestive process (Chae and Han 1998; Blasel, Hoffman, and Shaver 2006). However, too fine grinding may lead to increased stomach ulcers (Kiarie and Mills 2019).

Feed processing and enzyme supplementation have been identified as useful methods for enhancing digestive utilization of nutrients in NSP‐rich feedstuffs (Zijlstra, Owusu‐Asiedu, and Simmins 2010; de Vries et al. 2012). The combined application of these methods has emerged as a viable strategy to improve sustainability in pig and poultry production systems (Schedle 2016). However, the possible effects of PS and multienzyme supplement (MES) on standardized ileal digestibility (SID) of amino acids (AA) in hybrid rye fed to growing pigs are yet to be elucidated. Therefore, the objective of this study was to determine the effects of hybrid rye PS and MES on the SID of AA in two hybrid rye varieties fed to growing pigs. It was hypothesized that reduced PS and dietary MES supplementation would increase the SID of AA in hybrid rye fed to growing pigs.

## 2 Materials and Methods

The animal use protocol was reviewed and approved by the University of Manitoba Animal Care Committee (AC11419). All pigs were raised according to the guidelines of the Canadian Council on Animal Care (CCAC 2009).

### 2.1 Experimental Animals and Diets

Eighteen barrows (initial BW of 18.2 ± 1.0 kg; TN70 × TN Tempo; Topigs Norsvin, Winnipeg, MB, Canada) were housed individually in pens (1.47 × 1.14 m) with plastic‐covered woven metal floors in a temperature‐controlled room (22 ± 2°C). Each pen was equipped with a nipple drinker to allow pigs have unlimited access to water. Each pig was fitted with a T‐cannula at the distal ileum as described by Nyachoti et al. (2002) and given a 14‐day recovery period before being assigned to dietary treatments.

Two hybrid rye varieties (Bono and Gatano) were provided by KWS (KWS Lochow GmbH, Bergen, Germany) and ground using a hammer mill fitted with 4.0‐ and 3.2‐mm screens to obtain coarse and fine PS, respectively. The MES preparation (supplemented at 60 g/MT of feed) was provided by Canadian Bio‐System Inc. (Superzyme Conc; Calgary, Alberta, Canada). The MES supplied 420 units of cellulase, 45 units of mannanase, 510 units of xylanase, 180 units of glucanase, 4800 units of amylase, 240 units of invertase and 390 units of protease per kilogram of complete diet. Experimental diets consisted of 92.9% finely or coarsely ground hybrid rye as the only source of AA that was fed with or without MES supplementation and an N‐free diet to estimate the non‐specific endogenous losses of AA at the distal ileum (Table 1). For each hybrid rye diet, a basal diet was mixed and then divided into two halves. One half served as the control, whereas the other half was supplemented with the MES preparation to give the treatment diet. All diets contained 0.3% titanium dioxide as an indigestible marker.

**Table 1:** Ingredient composition of the experimental diets.

	Bono^a^		Gatano^a^		
Item	Fine	Coarse	Fine	Coarse	N‐free
Ingredient, %					
Hybrid rye	92.9	92.9	92.9	92.9	—
Cornstarch	—	—	—	—	68.1
Sucrose	—	—	—	—	20.0
Soybean oil	3.0	3.0	3.0	3.0	2.5
Limestone	1.2	1.2	1.2	1.2	0.9
Monocalcium phosphate	1.2	1.2	1.2	1.2	1.8
Salt	0.4	0.4	0.4	0.4	0.4
Cellulose^b^	—	—	—	—	5.0
Titanium dioxide	0.3	0.3	0.3	0.3	0.3
Vitamin‐mineral premix^c^	1.0	1.0	1.0	1.0	1.0

^a^ The hybrid rye diets utilized either finely or coarsely ground Bono or Gatano as the only source of amino acids and were formulated either with or without the inclusion of multienzyme supplement.

^b^ International Fibre Corp., North Tonawanda, NY, USA.

^c^ Provided the following per kg of complete diet: vitamins A, 2200 IU; vitamin D_3_, 220 IU; vitamin E, 16.0 IU; vitamin K, 0.5 mg; thiamine, 1.0 mg; riboflavin, 3.5 mg; niacin, 30.0 mg; pantothenic acid, 10.0 mg; biotin 0.05 mg; folic acid, 0.3 mg; vitamin B_12_, 0.02 mg; Cu, 6.0 mg as copper sulphate; I, 0.14 mg as calcium iodate; Fe, 100.0 mg as ferrous sulphate; Mn, 4.0 mg as manganese oxide; Se, 0.3 mg as sodium selenite; Zn, 100.0 mg as zinc oxide.

### 2.2 Experimental Design and Sample Collection

The experiment was conducted as a replicated 9 × 3 incomplete Latin square design to give six observations per diet. Pigs were weighed at the beginning of each period, with the average BW at the final period being 36.6 kg. Pigs were offered their daily ration as dry mash equivalent to 4% of BW divided into equal amounts at 0800 and 1600 h (Kiarie and Nyachoti 2007). Water was available at all times throughout the experiment. Each period lasted for 9 days, with the first 5 days serving as the adaptation period, followed by 2 days for faecal sample collection and the last 2 days for ileal digesta sample collection. Faecal samples were collected from each pen between 0800 and 1700 h on Days 6 and 7. Ileal digesta samples were collected continuously for 12 h (0800–2000 h) on Days 8 and 9, as described by Nyachoti et al. (2002). Digesta were collected in plastic bags attached to the barrel of the T‐cannula by a hose clamp. Digesta collection bags contained 10 mL of 10% (vol/vol) formic acid to minimize bacterial activity. Faecal and digesta samples were immediately frozen at −20°C until further processing.

### 2.3 PS Analysis, Sample Preparation and Chemical Analysis

Hybrid rye PS distribution was measured by using a sieve shaker (RX‐29, Superior Agri‐System, MB, Canada) fitted with 13 multiple sieves (US standard sieve Nos. 6, 8, 12, 16, 20, 30, 40, 50, 70, 100, 140, 200 and 270). In general, 150 g ground hybrid rye samples were sieved for 10 min and the geometric mean diameter (*D*_gw_), geometric standard deviation (*S*_gw_) and surface area were calculated according to the procedure of ANSI/ASAE S319.2 (American Society of Agricultural and Biological Engineers [ASABE] 1995).

Ileal digesta samples were thawed and pooled for each pig and period, homogenized in a blender (Waring Commercial, Torrington, CT, USA), subsampled and freeze‐dried. Faecal samples were dried in an oven at 50°C for 5 days, pooled per pig within a period and subsampled. Dried digesta, faeces and feed samples were ground to pass through a 1‐mm screen using a Foss Sample preparation Cyclotec 1093 mill (Foss Allé l, DK‐3400 Hilleroed, Denmark) before chemical analysis. Ingredients and diets samples were analysed for dry matter (DM), nitrogen (N), AA, ether extract (EE), gross energy (GE), neutral detergent fibre assayed with a heat stable amylase and expressed inclusive of residual ash (NDF), acid detergent fibre expressed inclusive of residual ash (ADF) and ash, with ingredients additionally analysed for calcium and phosphorus and diets additionally analysed for titanium dioxide. Faecal samples were analysed for DM, GE, N, EE, ash, NDF, ADF and titanium dioxide, while ileal digesta samples were analysed for DM, N, AA and titanium dioxide. Briefly, DM was determined according to procedure 934.01 of the Association of Official Analytical Chemists (AOAC International 2006). Nitrogen was analysed using a combustion analyzer (Model CNC‐2000; LECO Corporation, St. Joseph, MI, USA) and crude protein (CP) content was calculated as N × 6.25. The AA profiles were determined according to procedure 982.30 E (a,b,c) of AOAC International (2006). Tryptophan was determined following alkaline hydrolysis according to procedure 988.15 of AOAC International (2006). The GE content was measured using an isoperibol bomb calorimeter (model 6400, Parr Instrument Co., Moline, IL, USA) using benzoic acid as the calibration standard. The EE content was determined after hexane extraction according to procedure 920.39 of AOAC International (2006). The ash content was determined according to procedure 942.05 of AOAC International (2006). The concentrations of calcium and phosphorus were analysed according to procedure 968.08 and 946.06 of AOAC International (2006), respectively, and read on a Varian inductively coupled plasma mass spectrometer (Varian Inc., Palo Alto, CA, USA). The concentration of titanium dioxide was determined according to procedures described by Lomer et al. (2000) and read on an inductively coupled plasma mass spectrometer (Varian Inc., Palo Alto, CA, USA). The ADF and NDF contents were analysed according to the method of Goering and Van Soest (1970) using Ankom 200 Fibre Analyzer (ANKOM Technology, Macedon, NY, USA).

### 2.4 Calculations and Statistical Analysis

Apparent ileal digestibility (AID) of AA were calculated using the following equation (Stein et al. 2007):

$${\text{AID}}_{\text{AA}}\left(\%\right)=100-\left[\left({\text{AA}}_{d}/{\text{AA}}_{f}\right)\times \left({\text{Ti}}_{f}/{\text{Ti}}_{d}\right)\right]\times 100,$$

where AA_d_ is the AA content in ileal digesta (mg/kg DM), AA_f_ is the AA content in feed (mg/kg DM), Ti_f_ is the titanium dioxide concentration in feed (mg/kg DM) and Ti_d_ is the titanium dioxide concentration in ileal digesta (mg/kg DM). The AID of CP and apparent total tract digestibility (ATTD) values were also calculated using the same equation.

The non‐specific endogenous losses of AA (EAL) was measured at the distal ileum by feeding an N‐free diet to growing pigs and calculated according to the following equation:

$${\text{EAL}=\text{AA}}_{d}\times \left({\text{Ti}}_{f}/{\text{Ti}}_{d}\right),$$

where AA_d_ is the AA content in ileal digesta, Ti_f_ is the titanium dioxide concentration in feed (mg/kg DM) and Ti_d_ is the titanium dioxide concentration in ileal digesta (mg/kg DM). The non‐specific endogenous losses of CP was calculated using the same equation.

The SID values of AA were calculated using the following equation:

$${\text{SID}}_{\text{AA}}\left(\%\right)={\text{AID}}_{\text{AA}}+\left({\text{EAL}/\text{AA}}_{f}\right)\times 100,$$

where AID_AA_ represents the AID of AA; EAL is the non‐specific endogenous losses of AA (mg/kg DM) and AA_f_ is the AA content in feed (mg/kg DM). The SID of CP was calculated using the same equation.

Data were verified by the UNIVARIATE procedures of SAS 9.4 (SAS Inst. Inc., Cary, NC, USA) for outliers, and two outliers were detected since they were greater than 1.5 times the interquartile range and removed from the data set. Data were then analysed using the MIXED procedure of SAS. The effects of pen and period were not significant in the study, so they were not included in the final model. The final model had factors that included variety, PS, MES supplementation, and two‐ and three‐way interactions. The LSMEANS procedure was used to calculate mean values, and the PDIFF option was used to separate means. Results were considered significant at *p* < 0.05 and considered a trend at 0.05 < *p* < 0.10.

## 3 Results

All pigs readily consumed their feed throughout the study. The *D*_gw_ of coarsely and finely ground Bono were 1111 and 594 μm, and the respective values for coarsely and finely ground Gatano were 1210 and 717 μm (Table 2). Bono had greater surface area when finely ground compared with coarsely ground (128.7 vs. 60.7 cm^2^/g), and a similar pattern was observed in Gatano (98.4 vs. 51.3 cm^2^/g). As stated in Table 3, the CP content was slightly greater in Bono (9.31%) than in Gatano (9.19%). In hybrid rye, Leu (0.58%) was the most abundant indispensable AA, whereas Trp (0.08%) was the least abundant indispensable AA. The analysed nutrient composition of the experimental diets was displayed in Table 4.

**Table 2:** Particle size analysis of hybrid rye.^a^

Hybrid rye variety	Bono		Gatano	
Particle size	Fine	Coarse	Fine	Coarse
*Distribution of particles (%)*				
Sieve opening (μm)				
2360	0.60	1.33	0.73	1.36
1700	2.34	44.82	3.74	45.94
1180	23.95	17.67	30.29	20.30
850	23.21	12.89	25.88	13.78
600	14.98	7.35	13.14	6.49
420	9.10	4.14	7.14	3.24
297	6.15	3.09	4.67	2.40
212	4.88	2.01	3.60	1.48
150	3.08	1.61	2.13	1.20
103	2.27	1.08	1.73	0.88
73	1.07	0.84	0.87	0.72
53	1.20	0.32	1.07	0.48
37	7.16	2.85	5.00	1.72
Coarse particles (≥ 1700 μm)	2.94	46.14	4.47	47.30
Medium particles (297–1700 μm)	77.39	45.14	81.12	46.22
Fine particles (< 297 μm)	19.67	8.71	14.41	6.49
Geometric mean particle size (μm)	594	1111	717	1210
Standard deviation (μm)	2.77	2.43	2.55	2.20
Surface area (cm^2^/g)	128.7	60.7	98.4	51.3

^a^ Two hybrid rye varieties (Bono and Gatano) were ground using a hammer mill fitted with 4.0 and 3.2‐mm screens to obtain fine and coarse particle size, respectively.

**Table 3:** Analysed dnutrient composition of hybrid rye, as‐fed basis.

Variety	Hybrid rye^a^	
	Bono	Gatano
Analysed composition		
DM (%)	89.73	89.57
GE (MJ/kg)	16.15	16.09
CP (%)	9.31	9.19
EE (%)	1.18	1.42
Ash (%)	1.36	1.41
NDF (%)	11.24	13.35
ADF (%)	2.90	3.12
Calcium (%)	0.07	0.07
Phosphorus (%)	0.25	0.25
Indispensable AA (%)		
Arg	0.45	0.44
His	0.21	0.21
Ile	0.34	0.33
Leu	0.59	0.57
Lys	0.41	0.40
Met	0.17	0.16
Phe	0.41	0.41
Thr	0.31	0.30
Trp	0.08	0.07
Val	0.45	0.45
Dispensable AA (%)		
Ala	0.42	0.41
Asp	0.69	0.69
Cys	0.22	0.19
Glu	2.01	2.00
Gly	0.42	0.42
Pro	0.77	0.81
Ser	0.36	0.35
Tyr	0.14	0.14

Abbreviations: AA, amino acids; ADF, acid detergent fibre expressed inclusive of residual ash; CP, crude protein; DM, dry matter; EE, ether extract; GE, gross energy; NDF, neutral detergent fibre assayed with a heat stable amylase and expressed inclusive of residual ash.

^a^ Two hybrid rye varieties (Bono and Gatano) were finely ground with grain miller for 50–200 μm of fitness for chemical analysis.

**Table 4:** Analysed nutrient composition of the experimental diets, as‐fed basis.

Item	Bono^a^		Gatano^a^		N‐free
	Fine	Coarse	Fine	Coarse	
DM (%)	90.77	90.65	90.22	90.09	92.45
GE (MJ/kg)	16.80	16.69	16.87	16.55	15.66
CP (%)	8.32	8.79	7.85	7.94	0.63
EE (%)	6.25	5.52	6.52	5.72	1.68
Ash (%)	4.38	4.57	4.39	4.55	1.83
NDF (%)	11.49	11.38	12.46	12.38	—
ADF (%)	2.78	2.82	3.07	3.03	—
Indispensable AA (%)					
Arg	0.44	0.42	0.40	0.41	0.01
His	0.19	0.19	0.18	0.17	0.00
Ile	0.31	0.30	0.28	0.27	0.01
Leu	0.54	0.52	0.50	0.49	0.03
Lys	0.38	0.37	0.34	0.35	0.02
Met	0.15	0.15	0.13	0.13	0.01
Phe	0.38	0.36	0.35	0.34	0.02
Thr	0.29	0.28	0.26	0.26	0.01
Trp	0.08	0.07	0.07	0.07	0.02
Val	0.43	0.41	0.39	0.38	0.01
Dispensable AA (%)					
Ala	0.39	0.38	0.36	0.36	0.01
Asp	0.65	0.63	0.60	0.60	0.02
Cys	0.20	0.19	0.17	0.17	0.00
Glu	1.83	1.69	1.66	1.56	0.02
Gly	0.39	0.38	0.36	0.37	0.01
Pro	0.71	0.65	0.66	0.62	0.04
Ser	0.33	0.31	0.30	0.29	0.01
Tyr	0.18	0.16	0.15	0.17	0.01

Abbreviations: AA, amino acids; ADF, acid detergent fibre expressed inclusive of residual ash; CP, crude protein; DM, dry matter; EE, ether extract; GE, gross energy; N, nitrogen; NDF, neutral detergent fibre assayed with a heat stable amylase and expressed inclusive of residual ash.

^a^ The hybrid rye diets utilized either finely or coarsely ground Bono or Gatano as the only source of amino acids and were formulated either with or without the inclusion of multienzyme supplement.

There were no two‐ or three‐way interaction effects for the ATTD of energy and nutrients in hybrid rye diets (Table 5). The ATTD of DM, GE and CP was greater (*p* < 0.05) in Bono diets than in Gatano diets. The ATTD of NDF and EE was greater (*p* < 0.05) in finely ground hybrid rye diets than in coarsely ground hybrid rye diets, and the ATTD of GE tended to be greater (*p* = 0.077) in finely ground hybrid rye diets than in coarsely ground hybrid rye diets. Dietary MES supplementation increased (*p* < 0.05) the ATTD of DM, GE, CP, NDF, ADF and ash in hybrid rye diets.

**Table 5:** Effects of particle size (PS) and multienzyme supplement (MES) on apparent total tract digestibility (ATTD) of energy and nutrients in hybrid rye diets fed to growing pigs.

Treatments			ATTD (%)						
Variety	PS	MES	DM	GE	CP	NDF	ADF	EE	Ash
Bono	Fine	With	85.9	85.5	68.0	69.8	47.8	74.1	55.2
Bono	Fine	Without	84.2	83.9	63.8	63.4	40.5	69.4	48.4
Bono	Coarse	With	84.7	84.4	65.2	66.1	47.2	70.4	53.0
Bono	Coarse	Without	84.3	83.7	66.5	64.4	34.1	68.2	52.6
Gatano	Fine	With	85.2	85.0	66.4	69.4	47.7	76.8	57.5
Gatano	Fine	Without	83.2	82.9	61.5	65.6	38.8	73.9	53.7
Gatano	Coarse	With	84.0	83.4	63.0	63.1	43.0	69.9	56.5
Gatano	Coarse	Without	82.1	81.7	55.6	60.9	36.5	68.7	50.5
		SEM	0.725	0.777	2.165	2.221	3.135	2.857	2.796
*p‐*value									
Variety			0.031	0.048	0.008	0.464	0.684	0.374	0.276
PS			0.110	0.077	0.128	0.036	0.123	0.042	0.781
MES			0.006	0.031	0.018	0.030	< 0.001	0.184	0.045
Variety × PS			0.585	0.451	0.142	0.332	0.997	0.377	0.453
Variety × MES			0.382	0.506	0.137	0.743	0.570	0.729	0.741
PS × MES			0.545	0.554	0.642	0.191	0.693	0.599	0.603
Variety × PS × MES			0.546	0.829	0.201	0.627	0.368	0.922	0.295

Abbreviations: ADF, acid detergent fibre expressed inclusive of residual ash; CP, crude protein; DM, dry matter; EE, ether extract; GE, gross energy; NDF, neutral detergent fibre assayed with a heat stable amylase and expressed inclusive of residual ash.

The AID of CP and most AA in Gatano had greater effects of MES than Bono (Variety × MES; *p* < 0.05) (Tables 6 and 7). However, no other two‐way interactions or three‐way interactions were observed for the AID of CP and AA except Trp in hybrid rye. The AID values of CP and AA such as Met, Trp and Cys, were greater (*p* < 0.05) in Bono than in Gatano, and Bono tended to contain greater (*p* < 0.10) AID for Ile, Thr, Gly, Ser and Tyr than Gatano. There were no effects of PS on the AID of CP and AA in hybrid rye, with the exception that finely grinding tended to increase (*p* = 0.076) the AID of Tyr. Dietary MES supplementation increased (*p* < 0.05) the AID of CP and all AA, although it showed a tendency to increase (*p* = 0.096) the AID of Tyr.

**Table 6:** Effects of particle size (PS) and multienzyme supplement (MES) on apparent ileal digestibility (AID) of crude protein (CP) and indispensable amino acids in hybrid rye fed to growing pigs.

Treatments			AID (%)										
Variety	PS	MES	CP	Arg	His	Ile	Leu	Lys	Met	Phe	Thr	Trp	Val
Bono	Fine	With	71.0	76.9	77.3	77.2	77.4	70.4	83.0	79.8	69.5	74.4	75.8
Bono	Fine	Without	67.5	73.8	72.3	72.4	72.8	65.7	79.1	75.9	63.7	69.4	71.8
Bono	Coarse	With	69.4	72.4	72.0	73.4	73.3	64.6	78.5	76.3	64.5	75.2	72.2
Bono	Coarse	Without	68.9	74.0	73.5	73.4	73.4	64.5	80.0	76.1	65.1	73.3	72.4
Gatano	Fine	With	70.2	76.7	77.0	75.8	76.4	70.3	80.7	79.5	66.2	75.8	75.4
Gatano	Fine	Without	58.2	69.9	68.7	68.1	69.0	60.2	74.5	72.2	58.5	61.1	67.3
Gatano	Coarse	With	70.2	74.6	76.7	76.0	76.6	69.7	79.9	79.8	67.5	71.8	75.1
Gatano	Coarse	Without	60.7	70.3	69.6	67.7	69.7	56.4	74.1	72.9	59.2	68.5	67.9
		SEM	1.954	1.796	1.628	1.685	1.556	2.697	1.420	1.404	2.265	1.872	1.603
*p*‐value													
Variety			0.003	0.281	0.517	0.070	0.248	0.257	0.007	0.351	0.085	0.007	0.152
PS			0.674	0.240	0.437	0.536	0.556	0.143	0.244	0.588	0.809	0.137	0.539
MES			< 0.001	0.018	< 0.001	< 0.001	< 0.001	< 0.001	0.001	< 0.001	< 0.001	< 0.001	< 0.001
Variety × PS			0.325	0.603	0.302	0.587	0.341	0.739	0.530	0.270	0.387	0.794	0.464
Variety × MES			0.003	0.063	0.014	0.023	0.033	0.019	0.020	0.016	0.106	0.044	0.015
PS × MES			0.600	0.171	0.106	0.391	0.235	0.847	0.159	0.311	0.374	0.009	0.273
Variety × PS × MES			0.917	0.663	0.248	0.258	0.338	0.312	0.219	0.425	0.285	0.123	0.481

**Table 7:** Effects of particle size (PS) and multienzyme supplement (MES) on apparent ileal digestibility (AID) of dispensable amino acids in hybrid rye fed to growing pigs.

Treatments			AID (%)						
Variety	PS	MES	Ala	Asp	Cys	Glu	Gly	Ser	Tyr
Bono	Fine	With	71.7	76.3	77.8	86.0	52.6	73.5	71.9
Bono	Fine	Without	67.1	71.2	74.0	82.8	48.8	69.9	67.7
Bono	Coarse	With	66.6	71.8	74.7	84.4	52.1	71.0	70.1
Bono	Coarse	Without	66.8	72.2	76.4	84.2	51.3	70.6	68.0
Gatano	Fine	With	69.2	74.9	78.4	85.8	52.4	72.7	71.8
Gatano	Fine	Without	61.7	68.0	70.6	79.7	39.8	64.1	66.5
Gatano	Coarse	With	70.4	74.7	77.2	86.5	50.4	74.1	63.8
Gatano	Coarse	Without	61.1	67.3	69.0	80.7	40.3	65.1	65.3
		SEM	2.221	1.700	1.293	0.999	4.491	1.654	2.099
*p*‐value									
Variety			0.126	0.180	0.039	0.100	0.090	0.063	0.093
PS			0.456	0.360	0.333	0.609	0.974	0.887	0.076
MES			0.002	< 0.001	< 0.001	< 0.001	0.039	< 0.001	0.096
Variety × PS			0.358	0.572	0.580	0.490	0.784	0.382	0.205
Variety × MES			0.058	0.052	< 0.001	0.005	0.164	0.006	0.687
PS × MES			0.651	0.303	0.174	0.283	0.668	0.547	0.142
Variety × PS × MES			0.297	0.218	0.110	0.347	0.965	0.450	0.443

Likewise, the SID of CP and most AA in Gatano had greater effects of MES than Bono (Variety × MES; *p* < 0.05) (Tables 8 and 9). No additional two‐way interactions or three‐way interactions were observed for the SID of CP and AA in hybrid rye. The SID for CP was greater (*p* = 0.044) in Bono than in Gatano. However, neither the variety nor PS had an effect on the SID values for AA in hybrid rye, with the only exception that Bono had a higher (*p* = 0.026) SID of Met than Gatano. The addition of MES increased (*p* < 0.05) the SID of CP and all AA, except that it tended to increase (*p* = 0.060) the SID of Tyr.

**Table 8:** Effects of particle size (PS) and multienzyme supplement (MES) on standardized ileal digestibility (SID) of crude protein (CP) and indispensable amino acids (AA) in hybrid rye fed to growing pigs.^a^

Treatments			SID (%)										
Variety	PS	MES	CP	Arg	His	Ile	Leu	Lys	Met	Phe	Thr	Trp	Val
Bono	Fine	With	91.4	88.9	84.8	84.8	85.3	82.8	87.1	83.6	84.2	86.7	84.0
Bono	Fine	Without	88.4	86.8	80.2	80.3	81.1	78.4	83.5	83.1	78.9	81.6	80.2
Bono	Coarse	With	89.7	84.7	79.8	81.0	81.4	77.3	82.8	83.3	79.2	85.9	80.4
Bono	Coarse	Without	87.6	85.5	80.5	80.6	80.9	76.3	83.8	82.5	78.8	84.0	80.0
Gatano	Fine	With	91.6	89.5	85.3	84.2	85.1	83.7	85.3	86.8	82.5	88.0	84.2
Gatano	Fine	Without	80.2	82.5	77.1	76.6	77.5	73.2	79.1	79.5	74.3	75.4	76.1
Gatano	Coarse	With	91.5	88.2	84.6	84.2	84.9	83.0	84.6	86.8	83.2	86.0	83.6
Gatano	Coarse	Without	82.3	82.8	78.0	76.1	78.2	69.7	78.8	80.3	75.0	80.7	76.6
		SEM	1.953	1.798	1.627	1.687	1.551	2.695	1.419	1.404	2.264	1.873	1.604
*p‐*value													
Variety			0.044	0.582	0.902	0.236	0.496	0.494	0.026	0.590	0.347	0.130	0.368
PS			0.939	0.201	0.332	0.400	0.401	0.134	0.215	0.425	0.556	0.357	0.397
MES			< 0.001	0.010	< 0.001	0.001	0.001	< 0.001	< 0.001	< 0.001	0.001	< 0.001	< 0.001
Variety × PS			0.582	0.383	0.307	0.532	0.289	0.645	0.492	0.258	0.317	0.755	0.400
Variety × MES			0.008	0.037	0.021	0.030	0.035	0.021	0.024	0.022	0.104	0.046	0.021
PS × MES			0.418	0.366	0.150	0.446	0.299	0.947	0.225	0.385	0.456	0.056	0.335
Variety × PS × MES			0.816	0.807	0.415	0.355	0.514	0.427	0.299	0.628	0.452	0.428	0.622

^a^ Basal ileal endogenous losses for calculating SID of CP and indispensable AA were determined (mg/kg dry matter intake) as nitrogen, 3,019; arginine, 571; histidine, 156; isoleucine, 253; leucine, 461; lysine, 505; methionine, 67; phenylalanine, 277; threonine, 454; tryptophan, 95; valine, 370.

**Table 9:** Effects of particle size (PS) and multienzyme supplement (MES) on standardized ileal digestibility (SID) of dispensable amino acids (AA) in hybrid rye fed to growing pigs.^a^

Treatments			SID (%)						
Variety	PS	MES	Ala	Asp	Cys	Glu	Gly	Ser	Tyr
Bono	Fine	With	83.7	85.7	84.7	90.1	86.8	85.9	83.9
Bono	Fine	Without	79.4	85.1	81.2	87.2	83.9	82.7	81.3
Bono	Coarse	With	78.7	81.5	81.5	88.5	87.2	83.0	81.4
Bono	Coarse	Without	78.2	81.2	82.5	87.9	83.7	81.9	79.9
Gatano	Fine	With	82.1	85.0	85.9	90.3	88.2	85.9	84.4
Gatano	Fine	Without	74.4	78.0	78.2	84.3	74.7	77.3	78.4
Gatano	Coarse	With	83.0	84.7	84.8	90.6	86.1	86.4	79.4
Gatano	Coarse	Without	73.7	77.4	77.0	85.1	76.0	78.3	77.9
		SEM	2.222	1.699	1.292	0.998	4.493	1.653	2.101
*p*‐value									
Variety			0.283	0.375	0.281	0.233	0.201	0.230	0.293
PS			0.340	0.297	0.262	0.647	0.964	0.654	0.119
MES			0.001	< 0.001	< 0.001	< 0.001	0.024	< 0.001	0.060
Variety × PS			0.314	0.494	0.884	0.478	0.942	0.279	0.770
Variety × MES			0.058	0.058	0.001	0.007	0.185	0.012	0.570
PS × MES			0.722	0.401	0.233	0.330	0.831	0.595	0.337
Variety × PS × MES			0.398	0.346	0.223	0.533	0.766	0.732	0.581

^a^ Basal ileal endogenous losses for calculating SID of dispensable AA were determined (mg/kg dry matter intake) as alanine, 504; aspartic acid, 664; cysteine, 143; glutamic acid, 782; glycine, 1,430; serine, 425; tyrosine, 224.

## 4 Discussion

Hybrid rye has gained attention as a promising feed ingredient for pigs, especially due to its reduced ergot alkaloid levels and higher yield (Miedaner and Geiger 2015). The CP (9.25%) and AA contents in hybrid rye used in this study were lower than those reported by NRC (2012) and Rodehutscord et al. (2016) but within the range of values reported by McGhee and Stein (2018) and Strang et al. (2016), indicating that different rye cultivars may cause variations in protein and AA contents. Furthermore, this study identified compositional variance between the Bono and Gatano hybrid rye varieties, most notably in NDF content (11.24% vs. 13.35%), which might be attributed to genetic differences. The AID and SID of AA in hybrid rye, as determined in this study, were comparable to the values reported by NRC (2012). However, they were greater than the values reported by McGhee and Stein (2018) and Strang et al. (2016). This variance in AID and SID of AA could be attributed to several factors, including the specific genotype of the rye used, the overall composition of the diet with particular emphasis on the levels of dietary fibre and antinutritional components, the type of the indigestible marker used, and the processing methods applied. The EAL values determined in the current study were consistent with the range of values reported previously (Park, Oh, and Kim 2013). The BW of growing pigs can impact nutrient digestibility due to physiological and microbial changes in their digestive system as they mature (Huang et al. 2015). While BW might influence nutrient digestibility, our study's primary focus was on evaluating the treatments' effects on nutrient digestibility.

The grinding process holds a crucial role in feed manufacturing, serving to diminish PS while enhancing the digestibility of energy and nutrients in pigs (Rojas and Stein 2015). However, too fine PS is associated with an increased risk of gastric ulcers in pigs (Kiarie and Mills 2019). Therefore, the recommended PS for ground cereal grains is usually between 500 and 700 μm (Behnke 1994). Nonetheless, the use of fine grinding can lead to higher electrical energy usage and thus elevate the overall manufacturing costs for feed (Koo and Nyachoti 2021). Also, dietary multienzyme supplementation has been shown to improve energy and nutrients digestibility in various feedstuffs fed to pigs (Omogbenigun, Nyachoti, and Slominski 2004; Emiola et al. 2009). However, to the best of our knowledge, no study has yet explored its effect on AA digestibility in hybrid rye. Moreover, there has been a lack of research concerning how PS affects AA digestibility in hybrid rye. Therefore, this study was conducted to investigate the effects of both PS and MES supplementation on AA digestibility in hybrid rye fed to growing pigs.

In the current study, a major difference in PS between fine and coarse Bono was the substantially lower proportion of coarse particles (≥ 1700 μm) in the finely ground Bono (2.94%), compared to 46.14% in its coarse counterpart. A comparable pattern was also observed for Gatano, with 4.47% coarse particles (≥ 1700 μm) in the finely ground Gatano and 47.30% in the coarsely ground Gatano. When compared to coarse grinding, fine grinding substantially enlarged the surface area of the hybrid rye. For Bono, the surface area increased by 112% when fine grinding was applied, as opposed to coarse grinding. Similarly, fine grinding led to a 92% increase in the surface area for Gatano when compared to its coarsely ground counterpart.

Compared with Bono diets, Gatano diets showed lower ATTD of nutrients in terms of DM, GE and CP, which could be due to the greater NDF content in Gatano. Previous studies have suggested that higher NDF concentrations can adversely affect the DM and GE digestibility and that greater concentrations of soluble dietary fibre can increase viscosity and reduce nutrient digestibility (Annison and Choct 1991; Kim and Nyachoti 2017). Additionally, reducing the PS in hybrid rye diets increased the ATTD of NDF and EE and tended to increase the ATTD of GE, which could be explained by the fact that reduced PS increases the surface area of feed ingredients, thus allowing for better contact with the pig's digestive enzymes (Wondra et al. 1995). Moreover, the inclusion of MES in hybrid rye diets increased the ATTD of DM, GE, CP, NDF, ADF and ash, demonstrating that MES supplementation can break down NSP in hybrid rye, leading to better digestibility of an array of nutrients. The results of this study were in line with previous studies, such as those by Agyekum et al. (2016) and Huang et al. (2021), which demonstrated that multienzyme supplementation could significantly improve the ATTD of nutrients in diverse feed compositions. Similarly, enhancements in nutrient digestibility with enzyme supplementation were also noted by Kiarie et al. (2020) in roasted full‐fat soybeans and expelled‐extruded soybean meal diets and Niu et al. (2022) in diets high in canola meal.

No differences were found between Bono and Gatano regarding the SID of AA, which was consistent with the observations by McGhee and Stein (2018), who reported that the SID of AA in three varieties of rye was similar, and by Strang et al. (2016), who found no differences in SID of AA among eight rye genotypes, except for Cys. However, Bono did exhibit a higher SID of CP compared to Gatano, possibly due to Gatano's higher fibre content. Additionally, reducing PS did not increase the SID of AA in hybrid rye, a finding that was in agreement with the study by Rojas and Stein (2015), where corn PS did not affect the SID of AA. However, studies with other ingredients such as distillers dried grains with solubles and lupins have shown that reducing PS can increase the SID of AA (Kim et al. 2009; Yáñez et al. 2011). These inconsistencies might be attributed to the impact of grinding intensity on the flow behaviour of the digesta between hybrid rye and other ingredients.

Dietary MES supplementation increased the SID of AA in hybrid rye, which was consistent with the previous study by Ayoade et al. (2012), in which multienzyme supplementation increased the SID of AA, such as Leu, Met + Cys, Thr, Cys, Ser and Tyr in extruded full‐fat soybeans. However, Dadalt et al. (2016) reported that multienzyme supplementation did not affect most AA digestibility in pea protein isolate for growing pigs. Similarly, a study by Kiarie et al. (2010) indicated that multienzyme supplementation did not affect the digestibility of most AA in a wheat and barley‐based diet fed to growing pigs. These different outcomes could be due to hybrid rye's higher NSP content compared to the other ingredients. Interestingly, this study also revealed interactions between variety and MES supplementation on the SID of CP and most AA. This might be because MES was more effective on Gatano than on Bono, potentially due to Gatano's higher fibre content.

## 5 Conclusion

The digestibility of nutrients in hybrid rye is influenced by the variety used. Reducing the hybrid rye PS enhanced nutrient digestibility, whereas dietary supplementation of MES improved the digestibility of energy and AA in hybrid rye diets for growing pigs. Notably, interactions between rye variety and MES were observed, particularly affecting the ileal digestibility of CP and AA such as His, Ile, Leu, Lys, Met, Phe, Trp, Val, Cys, Glu and Ser. These findings suggest that both reducing PS and dietary supplementation of MES can enhance the nutritional value of hybrid rye for pigs.

## Author Contributions

**Jichen Song:** conceptualization, data curation, formal analysis, investigation, methodology, visualization, writing–original draft. **Claus H. Heuer** and **Rob Patterson:** resources, writing–review and editing. **Charles M. Nyachoti:** conceptualization, funding acquisition, supervision, project administration, resources, writing–review and editing.

## Ethics Statement

The authors confirm that the ethical policies of the journal, as noted on the journal's author guidelines page, have been adhered to and the appropriate ethical review committee approval has been received. The animal use protocol was reviewed and approved by the University of Manitoba Animal Care Committee (AC11419). The authors confirm that they have followed EU standards for the protection of animals used for scientific purposes.

## Conflicts of Interest

The authors declare no conflicts of interest.

## Supporting information

Supporting information.

## Data Availability

The data that support the findings of this study are available from the corresponding author upon reasonable request.
