# Supplementary material for: Standardized Ileal Digestibility of Amino Acids in Hybrid Rye Ground to Two Particle Sizes and Fed With or Without Multienzyme Supplement to Young Growing Pigs
Source: J Anim Physiol Anim Nutr (Berl). 2024 Oct 23;109(2):411–22. doi: 10.1111/jpn.14053 (PMC11919803; doi:10.1111/jpn.14053)
Supplement: Supplementary file 1 — Supporting information. [file JPN-109-411-s001.docx]

**Supplementary Table 1.** Non-specific endogenous losses of nitrogen (N) and amino acids (AA) at the distal ileum of growing pigs fed N-free diet (mg/kg of DM)

| Item | N-free diet |
| --- | --- |
| N | 3,019 |
| Indispensable AA |  |
| Arg | 571 |
| His | 156 |
| Ile | 253 |
| Leu | 461 |
| Lys | 505 |
| Met | 67 |
| Phe | 277 |
| Thr | 454 |
| Trp | 95 |
| Val | 370 |
| Dispensable AA |  |
| Ala | 504 |
| Asp | 664 |
| Cys | 143 |
| Glu | 782 |
| Gly | 1,430 |
| Ser | 425 |
| Tyr | 224 |
